# Exploring the Epidemiology of Atrial Fibrillation in Nigeria: A Focus on Atrial Fibrillation in Heart Failure Patients and Its Association With Stroke

**DOI:** 10.7759/cureus.55241

**Published:** 2024-02-29

**Authors:** Emmanuel O Otabor, Abiola Alaka, Chidera N Onwuzo, John Olukorode, Adeniyi Fagbenro, Adetayo Ajiboye

**Affiliations:** 1 Internal Medicine, South Warwickshire NHS Foundation Trust, Warwick, GBR; 2 Internal Medicine, University of Michigan, Ann Arbor, USA; 3 Internal Medicine, Benjamin S. Carson College of Health and Medical Sciences, Ilishan-Remo, NGA; 4 Internal Medicine, Bowen University College of Health Sciences, Iwo, NGA; 5 Dermatology, Central and Northwest London NHS, London, GBR

**Keywords:** cardiovascular disease, epidemiology of atrial fibrillation, quality of life, atrial fibrillation, heart failure, stroke

## Abstract

Atrial fibrillation (AF) represents a significant global public health concern, particularly due to its association with adverse health outcomes such as stroke and heart failure. In Nigeria, where the burden of cardiovascular diseases is rising, understanding the prevalence and impact of AF is crucial for effective healthcare planning and intervention strategies.

This review examines the epidemiology of AF in Nigeria, comparing it with global and African data. It explores demographic and regional variations, comorbidity factors, and the impact of AF on the healthcare system, mortality, and quality of life.

Notably, the prevalence of AF in Nigeria generally falls just under 5%, but this figure rises to approximately 9% in stroke patients and 11-20% among those with heart failure (HF). Rheumatic heart disease (RHD) is identified as a significant AF risk factor within Africa, affecting around 20% of AF patients - a stark contrast to the 2% in North America. AF's association with higher mortality rates and functional deterioration highlights the urgent need for improved diagnostic and therapeutic approaches, alongside broader public health measures.

In conclusion, the review emphasises the significant public health concern AF represents in Nigeria, especially among HF and stroke patients, and stresses the importance of tailored healthcare policies and interventions to mitigate AF's impact and improve patient outcomes.

## Introduction and background

Atrial fibrillation (AF) represents the most prevalent form of persistent cardiac arrhythmia and constitutes a major worldwide public health issue [1]. This condition is characterised by rapid and disorganised electrical activity in the atria, leading to ineffective contractions and an increased risk of thromboembolic events. The etiologies of AF are multifaceted, involving risk factors such as diabetes, hypertension, ageing, dyslipidemia, obesity, and smoking. Studies show that AF and coronary artery disease (CAD) often coexist due to these shared factors, leading to pathophysiological changes and a higher risk of either condition. Typically, as the prevalence of CAD risk factors increases, so does the AF likelihood [2,3].

The rising incidence of AF worldwide highlights its significant clinical impacts, including increased risks of stroke, heart failure, systemic embolism, and a growing link to cognitive decline and dementia, potentially due to microembolisms, bleeds, non-cortical/cortical infarcts, and chronic hypoperfusion. These factors collectively contribute substantially to morbidity and mortality [4,5]. In Nigeria, for example, the coexistence of AF and heart failure (HF) presents a troubling scenario for cardiovascular health. Studies indicate a high prevalence of AF among HF patients, leading to a worse prognosis and further deterioration in the quality of life [6,7]. This makes the diagnosis and treatment of AF and its cardiovascular complications a matter of considerable importance within the field of cardiology.

In many developing nations, including Nigeria, there has been a gradual shift in disease trends from infectious to non-communicable diseases. This epidemiological transition has also been driven by a surge in cardiovascular risk factors such as hypertension, obesity, and diabetes mellitus [8]. The rise in the prevalence of these risk factors in Nigeria and sub-Saharan Africa has significant implications for the incidence of AF and other cardiovascular conditions [9,10].

This review aims to comprehensively explore the epidemiology of atrial fibrillation in Nigeria, examining its prevalence and comparing it with global and African data. The study delves into demographic and regional variations, including age-specific epidemiology, gender differences, rural-urban disparities, and regional distinctions. Additionally, it investigates comorbidity factors associated with atrial fibrillation in the Nigerian population, considering lifestyle, cultural, socioeconomic, and environmental influences. Lastly, the study explores the impact of AF on the healthcare system, its economic complications, effects on quality of life, mortality, and healthcare accessibility in Nigeria. Understanding these critical issues can aid public health initiatives and strategic resource allocation aimed at improving patient outcomes.

## Review

The prevalence of atrial fibrillation: global and African perspectives

When compared to other regions, data on the prevalence and incidence of AF in Africa is limited. Globally, the overall incidence and prevalence of AF are increasing, with estimates reaching 46.3 million individuals worldwide in 2016, according to the Global Burden of Disease Project [11]. This growing burden is attributed to various factors such as the rise in detection and the ageing demographic.

Significant variations in AF prevalence have been observed in diverse studies across Nigeria. In Enugu, a hospital-based study at a tertiary centre analysed the electrocardiograms (ECGs) of 860 inpatients over five months, revealing a prevalence of 3.5% for AF [12]. Similarly, a study conducted at a suburban tertiary hospital in Southwest Nigeria from January 2012 to December 2015, involving 1462 patients, reported a 3.76% prevalence of AF [13].

Study results from Nigeria align with findings in other African countries [12,13]. Across Africa, hospital-based studies indicate an AF prevalence of around 5%, with specific country variations. For instance, in a study in South Africa, 4.6% of cardiology patients were found to have AF, representing an estimated prevalence of 5.6 cases per 100,000 populations per year [14]. Similarly, another study in Ivory Coast over a 10-year period reported a prevalence of 5.5% among admitted individuals. Mbaye et al. noted a prevalence of 5.4% in Senegal while Shavadia et al. in Nairobi, Kenya, reported 0.7% [15-17]. Notably, a community-based study in rural Ghana highlighted a significantly lower prevalence of 0.3% among individuals aged 50 and older [18]. However, it is critical to note that, in contrast to the community-based Ghanaian study, the aforementioned studies primarily involved inpatient settings, where the prevalence of AF may be inflated due to acute illnesses.

In the developed world, the prevalence of AF averages 1-2%, increasing with age. Projections for the United States suggest a surge to 12.1 million affected individuals by 2030 [19]. In Europe, the prevalence of AF was approximately 9 million in 2010, and it is anticipated to reach 14 million by 2060 among those aged over 55 years [20].

AF in heart failure patients

Atrial fibrillation and heart failure have emerged as notable cardiovascular epidemics in recent years, prompting research into their intricate pathophysiological relationship [21]. AF plays a role in the development or progression of heart failure through various mechanisms. Elevated resting heart rate and abnormal exercise response contribute to a shortened diastolic filling time, resulting in diminished cardiac output. The irregular ventricular response further compounds this effect, and the loss of effective atrial contractile function becomes particularly significant in individuals with diastolic dysfunction. Conversely, heart failure can predispose individuals to AF through multiple pathways. Elevated cardiac filling pressures, interstitial fibrosis, dysregulation of intracellular calcium, and dysfunction in autonomic and neuroendocrine systems are some of the implicated mechanisms [22].

The frequent association of atrial fibrillation (AF) with heart failure (HF) has raised awareness of its detrimental prognostic impact on the disease's natural progression. Like the prevalence of AF in the general population, there is a noticeable upward trend in patients with coexisting heart failure, reaching rates as high as 35% in previous reports [23,24]. Notably, studies conducted in Africa have reported lower rates compared to heart failure registries in other global regions. In the United States, Euro Heart Failure Survey II and Acute Decompensated Heart Failure Syndrome Registry of Asia, AF prevalence was reported at 31%, 39%, and 36%, respectively [25]. In contrast, The Sub-Saharan Africa Survey on HF (THESUS-HF) registry documented an 18% prevalence of AF among heart failure patients [26]. The IDAN-HF study sheds light on AF prevalence within the heart failure population in Nigeria, revealing a significant rate of 20% [7]. This data not only offers insights into the current state of AF in this patient group but also reflects broader epidemiological concerns within the Nigerian healthcare system, indicating a substantial clinical burden. Supporting these findings, the Abeokuta Heart Failure registry - a hospital-based, single-centre, prospective, observational study that enrolled 285 subjects with new-onset acute heart failure (AHF) over two years - recorded an 11.5% prevalence of AF among its participants [27].

Research in Kano, Nigeria, spanning 2005 to 2006, disclosed an AF prevalence of 14.1% in individuals diagnosed with heart failure with reduced left ventricular ejection fraction (HFrEF) and a higher rate of 19.1% among those with heart failure with preserved ejection fraction (HFpEF) [28]. In line with these findings, a separate investigation in a tertiary hospital in Kano reviewed the prevalence and clinical characteristics of heart failure patients with atrial fibrillation, identifying a prevalence of 12.5% within the examined heart failure group [29]. These findings align with previous studies, reinforcing the recognition of AF as a common comorbidity in heart failure patients.

In the context of Nigeria, the reported AF prevalence appears lower than that in the US and Europe, but higher than in some other African countries. Sliwa et al. reported a lower AF prevalence of 6.6% among 2393 HF patients in Soweto, South Africa, and a hospital-based study in Kumasi, Ghana, identified AF in 8.9% of HF patients [14,30].

AF and stroke

Stroke poses a significant global health challenge, ranking as one of the leading causes of death worldwide and exerting substantial impact, particularly in developing regions such as sub-Saharan Africa (SSA) [31]. The condition can be broadly classified into haemorrhagic and ischaemic types. However, studies in Africa face limitations in stroke diagnosis and subtype classification, leading to adverse outcomes. Factors contributing to these challenges include limited access to brain imaging and cardiovascular examinations such as ECG and echocardiography, the scarcity of functional stroke units, and the limited availability of neurologists [32]. These constraints are further intensified by patients' financial limitations, hindering their ability to afford essential diagnostic tests.

Stroke prevalence rates vary across Africa. In a 2007 study, the reported crude prevalence rate for stroke in urban Nigeria was 1.14/1,000, which is comparatively lower than rates observed in most developed countries [33]. Over the period from 2009 to 2013, there was a notable 10.8% increase in the incidence of stroke in Africa, primarily associated with the growth and ageing of the African population [34]. Insights derived from the INTERSTROKE study's epidemiological data disclose that in Africa, the proportions of ischaemic and haemorrhagic strokes are 66% and 34%, respectively [35]. Similarly, in Nigeria, a prospective cohort study conducted at the National Hospital Abuja, focusing on patients with acute stroke from January 2010 to June 2012, reported that 62% had ischaemic stroke while 32% experienced haemorrhagic stroke [36]. Findings from neighbouring Cameroon present a slight variation, with ischaemic strokes accounting for 52%, and haemorrhagic strokes for 48% [37]. These distributions contrast with high-income countries, where ischaemic strokes predominate at 91%, and haemorrhagic strokes at 9% [35].

AF stands out as a leading cause of cardioembolic stroke and is linked to significant morbidity and economic burden [38]. Recognizing AF as a cause of stroke is crucial, as anticoagulation measures can effectively prevent recurrent strokes [39]. In a cross-sectional study conducted in Nigeria, which scrutinised a cohort of 369 stroke patients, AF was identified in 9.6% of the participants. Initially, 27 individuals with suspected cardioembolic stroke exhibited unremarkable findings on ECGs. Subsequent evaluation was conducted through 24-hour Holter monitoring, revealing the presence of AF [40]. The SIREN (Stroke Investigative Research and Educational Network) study, initiated in August 2014 as a multicenter case-control investigation across diverse sites in Nigeria and Ghana, provided valuable insights into the association between AF and stroke. Within the initial 24 hours following acute ischaemic stroke, 60%-85% of patients exhibited ECG abnormalities, with AF being present in 4.2% of cases. Notably, patients with ischaemic stroke demonstrated a significantly higher likelihood of having AF compared to those with haemorrhagic stroke [41]. In a five-year analysis conducted by Ibrahim et al. to examine the clinical presentations and predictors of stroke mortality in rural Southwestern Nigeria. Among 309 patients confirmed by CT imaging to have experienced a stroke, the majority (90.2%) had a stroke for the first time. Atrial fibrillation was detected on ECG in 18.8% of the affected patients [42]. Alkali et al. in their study reported an AF prevalence of 9.2% among patients presenting at the National Hospital Abuja with acute stroke [36].

The observed prevalence of atrial fibrillation (AF) as a risk factor in stroke patients, as indicated by studies conducted in Nigeria, seems to surpass that reported in certain other African nations. In Sierra Leone and Malawi, for instance, a lower prevalence of 6.1% and 7%, respectively, was documented [43,44]. This difference may be attributed to the constrained availability of ECG at the time of admission in some of these studies [35,44].

Risk factors of AF

Atrial fibrillation (AF) has been associated with various risk factors, ranging from dietary and lifestyle factors to conditions such as hypertension, diabetes, and heart disease [45]. Notably, the Framingham Heart Study firmly established the connection between hypertension and AF, indicating that a blood pressure (BP) exceeding >160/95 mmHg is associated with an elevated AF risk, with odds ratios of 1.5 for men and 1.4 for women [46]. In line with this, a study conducted by Anisiuba et al. in Enugu, Nigeria, aimed at determining the incidence of AF, reported hypertension as the predominant underlying condition in 33.3% of cases, followed by valvular heart disease at 23.3% [12]. This evidence reinforces the correlation between hypertension and AF.

Type 2 diabetes mellitus has been identified as a significant contributor to the risk of new-onset AF, demonstrating a 1.5 times increased risk. Moreover, a dose-response relationship has been observed between blood glucose levels and AF risk in individuals with diabetes and pre-diabetes, with respective increases of 28% and 20% [47].

Lifestyle factors also play a crucial role in AF incidence. Larsson et al. reported a noteworthy association between moderate alcohol intake and AF, revealing a relative risk (RR) of 1.08 (95% CI: 1.06 to 1.10) even with as low as one drink per day [48]. Smoking not only increases the incidence of AF but also heightens the risk of thromboembolic events in AF patients [49]. Encouragingly, quitting smoking has been demonstrated to reduce the risk of AF by 36% [50]. Sedentary behaviour, implicated as a risk factor for various cardiovascular diseases, has been linked to AF through the development of insulin intolerance, leading to endothelial cell death and inflammation [51].

In the context of Africa, Rheumatic Heart Disease (RHD), particularly mitral stenosis, emerges as a significant risk factor for AF [45]. RHD is prevalent in 21% of AF patients [14], aligning with the findings of the Randomised Evaluation of Long-Term Anticoagulation Therapy (RE-LY) AF study, where RHD was present in 22% of African AF patients compared to 2% in North American patients [52]. It also tallies with the findings of Anisiuba et al. [12].

Sub-Saharan Africa exhibits distinctive patterns of comorbidities in AF patients, with hypertension prevalent in 50-87%, heart failure in 32-64%, diabetes in 4-63%, RHD in 15-38%, and dilated cardiomyopathy in 16-38% [53]. This contrasts with the Caucasian population, where coronary artery disease, congestive heart failure, and obesity are more predominant [54].

A study conducted in Port-Harcourt, Nigeria, identified hypertensive heart disease, dilated cardiomyopathies, and RHD as the most common comorbidities associated with AF [55]. Similarly, a Lagos-based study reported the prevalence of hypertensive heart disease at 46.2%, dilated cardiomyopathy at 20.8%, RHD at 17.9%, thyrotoxic heart disease at 7.7%, and hypertrophic cardiomyopathy at 5.1% [56].

Impact of atrial fibrillation

In Nigeria and other sub-Saharan African countries, the concurrent existence of infectious and non-communicable diseases presents a formidable challenge to the healthcare system [8]. One of the aims of this study was to elucidate the prevalence and impact of atrial fibrillation (AF), especially in stroke and heart failure (HF), contributing substantial data to enhance our comprehension of the evolving cardiovascular disease landscape in Nigeria. Our findings indicate that AF is not an infrequent occurrence among the Nigerian populace, with a prevalence slightly below 5% [12,13]. A significantly higher prevalence is observed among patients with heart failure, ranging between 11% and 20% [7,27-29]. The correlation of AF with the severity of HF, particularly in New York Heart Association (NYHA) class IV patients, highlights the interplay between these two conditions [7].

Research indicates that Africans with heart failure (HF) face a notably higher risk of death, exceeding twice the global average [57]. The presence of AF adds complexity to predicting outcomes for these patients by worsening their overall health conditions. According to Akintunde et al., the increased occurrence of AF among Heart Failure with Reduced Ejection Fraction (HFrEF) patients suggests a potentially serious situation, signalling a more severe prognosis and advanced symptoms of HF [7]. In the Nigerian context, the predominance of hypertension as a risk factor for AF demonstrates the need for focused diagnostics, interventions and awareness campaigns [12]. Analysing how AF affects the functional status and outlook of HF patients reveals that AF is associated with worse traditional indicators of health problems and death. These include lower ejection fraction, kidney issues, pulmonary hypertension, and thromboembolic risk. Patients with coexisting AF and HF are likely to experience a noticeable impact on their daily lives [7].

Patients diagnosed with atrial fibrillation (AF) exhibit an increased susceptibility to thromboembolic events, emphasising the necessity for anticoagulation therapy [29]. As previously highlighted with references to findings from the INTERSTROKE study, there is a significant elevation in the risk of ischaemic strokes linked with AF [35]. Despite the acknowledged correlation between AF and stroke, Sub-Saharan African countries exhibit suboptimal management practices characterised by low utilisation of anticoagulants [14,55]. The observed non-compliance with recommended anticoagulation therapy necessitates immediate attention to mitigate the associated risk of stroke.

There are limited data available on the financial impact of AF in Nigeria. Given the frequent co-occurrence of AF with heart failure and other serious conditions, such as stroke, it is reasonable to infer that its financial implications are significant. A notable study by Ogah et al. in Abeokuta, Nigeria, sheds light on the significant economic toll of heart failure on both individuals and the broader community. The research reveals that individuals affected by HF are relatively young, with an average age of 58. This fact, combined with the country's low minimum wage and the widespread reliance on out-of-pocket payments for healthcare, significantly amplifies the economic burden of HF on patients and society [58].

This review extensively explores atrial fibrillation in Nigeria, including its impact on heart failure patients, and highlighting its association with certain comorbidities, increased mortality, functional decline, and an elevated risk of stroke. These findings shed light on the cardiovascular disease landscape in the region, stressing the importance of targeted interventions.

## Conclusions

Collectively, these studies illustrate a clear and pressing epidemiological trend of AF among various cardiovascular patient groups in Nigeria. Such prevalence data are critical for the healthcare system to recognize the extent of the issue; however, the lack of incidence rates, which are essential for tracking the condition’s progression and forecasting future trends, indicates a gap in knowledge.

## References

[1] Benjamin EJ, Muntner P, Alonso A (2019). Heart disease and stroke statistics-2019 update: a report from the American Heart Association. Circulation.

[2] Batta A, Hatwal J, Batta A, Verma S, Sharma YP (2023). Atrial fibrillation and coronary artery disease: an integrative review focusing on therapeutic implications of this relationship. World J Cardiol.

[3] Mekhael M, Marrouche N, Hajjar AH, Donnellan E (2024). The relationship between atrial fibrillation and coronary artery disease: understanding common denominators. Trends Cardiovasc Med.

[4] Tang SC, Liu YB, Lin LY (2023). Association between atrial fibrillation burden and cognitive function in patients with atrial fibrillation. Int J Cardiol.

[5] Lane DA, Skjøth F, Lip GY, Larsen TB, Kotecha D (2017). Temporal trends in incidence, prevalence, and mortality of atrial fibrillation in primary care. J Am Heart Assoc.

[6] Batta A, Sharma YP, Hatwal J, Panda P, Kumar BG, Bhogal S (2022). Predictors of dementia amongst newly diagnosed non-valvular atrial fibrillation patients. Indian Heart J.

[7] Akintunde AA, Akinlade OM (2023). Atrial fibrillation in heart failure: epidemiology, quality of life and clinical characteristics in the Iron Deficiency and Anaemia in Heart Failure (IDAN-HF) study in Ogbomoso, Nigeria. Ethiop J Health Sci.

[8] (2018). WHO. Noncommunicable diseases country profiles. https://www.who.int/publications/i/item/9789241514620.

[9] Olamide O, Adebayo O, Emmanuel A, Eyitayo L, Beatrice O, Tomisin M (2022). Prevalence and risk factors of cardiovascular diseases among the Nigerian population: a new trend among adolescents and youths. Novel Pathogenesis and Treatments for Cardiovascular Disease.

[10] Kadiri S (2005). Tackling cardiovascular disease in Africa. BMJ.

[11] Benjamin EJ, Muntner P, Alonso A (2019). Heart disease and stroke statistics—2019 update: a report from the American Heart Association. Circulation.

[12] Anisiuba BC, Ejim EC, Onwubere BJC, Ikeh VO, Ike SO (2005). Atrial fibrillation cases seen at the University of Nigeria Teaching Hospital, Enugu. International Journal of Medicine and Health Development.

[13] Ajayi EA, Adeyeye VO, Adeoti AO (2016). Clinical and echocardiographic profile of patients with atrial fibrillation in Nigeria. J Health Sci.

[14] Sliwa K, Carrington MJ, Klug E, Opie L, Lee G, Ball J, Stewart S (2010). Predisposing factors and incidence of newly diagnosed atrial fibrillation in an urban African community: insights from the Heart of Soweto Study. Heart.

[15] Coulibaly I, Anzouan-Kacou JB, Konin KC, Kouadio SC, Abouo-N'Dori R (2010). Atrial fibrillation: epidemiological data from the Cardiology Institute in Abidjan, Côte d'Ivoire [Article in French]. Med Trop (Mars).

[16] Mbaye A, Pessinaba S, Bodian M (2010). Atrial fibrillation, frequency, etiologic factors, evolution and treatment in a cardiology department in Dakar, Senegal [Article in French]. Pan Afr Med J.

[17] Shavadia J, Yonga G, Mwanzi S, Jinah A, Moriasi A, Otieno H (2013). Clinical characteristics and outcomes of atrial fibrillation and flutter at the Aga Khan University Hospital, Nairobi. Cardiovasc J Afr.

[18] Koopman JJ, van Bodegom D, Westendorp RG, Jukema JW (2014). Scarcity of atrial fibrillation in a traditional African population: a community-based study. BMC Cardiovasc Disord.

[19] Colilla S, Crow A, Petkun W, Singer DE, Simon T, Liu X (2013). Estimates of current and future incidence and prevalence of atrial fibrillation in the U.S. adult population. Am J Cardiol.

[20] Krijthe BP, Kunst A, Benjamin EJ (2013). Projections on the number of individuals with atrial fibrillation in the European Union, from 2000 to 2060. Eur Heart J.

[21] Braunwald E (1997). Cardiovascular medicine at the turn of the millennium: triumphs, concerns, and opportunities. N Engl J Med.

[22] Anter E, Jessup M, Callans DJ (2009). Atrial fibrillation and heart failure: treatment considerations for a dual epidemic. Circulation.

[23] Hamaguchi S, Kinugawa S, Matsushima S (2014). Clinical characteristics and CHADS2 score in patients with heart failure and atrial fibrillation: insights from the Japanese Cardiac Registry of Heart Failure in Cardiology (JCARE-CARD). Int J Cardiol.

[24] Ozierański K, Kapłon-Cieślicka A, Peller M (2016). Clinical characteristics and predictors of one-year outcome of heart failure patients with atrial fibrillation compared to heart failure patients in sinus rhythm. Kardiol Pol.

[25] Damasceno A, Cotter G, Dzudie A, Sliwa K, Mayosi BM (2007). Heart failure in sub-Saharan Africa: time for action. J Am Coll Cardiol.

[26] Damasceno A, Mayosi BM, Sani M (2012). The causes, treatment, and outcome of acute heart failure in 1006 Africans from 9 countries. Arch Intern Med.

[27] Ogah OS, Stewart S, Falase AO (2014). Contemporary profile of acute heart failure in Southern Nigeria: data from the Abeokuta Heart Failure Clinical Registry. JACC Heart Fail.

[28] Karaye KM, Sani MU (2008). Electrocardiographic abnormalities in patients with heart failure. Cardiovasc J Afr.

[29] Ringim SH, Ibrahim UM, Hamza SM, Dankishiya FS, Mijinyawa MS, Sani MU (2023). Prevalence and clinical profile of heart failure patients with atrial fibrillation in a tertiary hospital in Kano, Nigeria. Niger Postgrad Med J.

[30] Owusu IK, Adu‑Boakye Y, Appiah LT (2014). Electrocardiographic abnormalities in heart failure patients at a teaching hospital in Kumasi, Ghana. J Cardiovasc Dis Diagn.

[31] Murray CJ, Lopez AD (1997). Mortality by cause for eight regions of the world: Global Burden of Disease Study. Lancet.

[32] Chin JH (2012). Stroke in sub-Saharan Africa: an urgent call for prevention. Neurology.

[33] Danesi M, Okubadejo N, Ojini F (2007). Prevalence of stroke in an urban, mixed-income community in Lagos, Nigeria. Neuroepidemiology.

[34] Adeloye D (2014). An estimate of the incidence and prevalence of stroke in Africa: a systematic review and meta-analysis. PLoS One.

[35] O'Donnell MJ, Xavier D, Liu L (2010). Risk factors for ischaemic and intracerebral haemorrhagic stroke in 22 countries (the INTERSTROKE study): a case-control study. Lancet.

[36] Alkali NH, Bwala SA, Akano AO, Osi-Ogbu O, Alabi P, Ayeni OA (2013). Stroke risk factors, subtypes, and 30-day case fatality in Abuja, Nigeria. Niger Med J.

[37] Mapoure YN, Kuate C, Tchaleu CB (2014). Stroke epidemiology in Douala: three years prospective study in a teaching hospital in Cameroon. World J Neurosci.

[38] Stewart S, Hart CL, Hole DJ, McMurray JJ (2002). A population-based study of the long-term risks associated with atrial fibrillation: 20-year follow-up of the Renfrew/Paisley study. Am J Med.

[39] Ruff CT, Giugliano RP, Braunwald E (2014). Comparison of the efficacy and safety of new oral anticoagulants with warfarin in patients with atrial fibrillation: a meta-analysis of randomised trials. Lancet.

[40] Alkali NH, Bwala SA, Dunga JA, Watila MM, Jibrin YB, Tahir A (2016). Prestroke treatment of stroke risk factors: a cross-sectional survey in central Nigeria. Ann Afr Med.

[41] Adeoye AM, Ogah OS, Ovbiagele B (2017). Prevalence and prognostic features of ECG abnormalities in acute stroke: findings from the SIREN study among Africans. Glob Heart.

[42] Ibrahim AO, Shabi OM, Agbesanwa TA, Olowoyo P (2022). Five-year analysis of clinical presentations and predictors of stroke mortality in rural Southwestern Nigeria: a retrospective observational study. Afr J Emerg Med.

[43] Russell JB, Charles E, Conteh V, Lisk DR (2020). Risk factors, clinical outcomes and predictors of stroke mortality in Sierra Leoneans: a retrospective hospital cohort study. Ann Med Surg (Lond).

[44] Heikinheimo T, Chimbayo D, Kumwenda JJ, Kampondeni S, Allain TJ (2012). Stroke outcomes in Malawi, a country with high prevalence of HIV: a prospective follow-up study. PLoS One.

[45] January CT, Wann LS, Alpert JS (2014). 2014 AHA/ACC/HRS guideline for the management of patients with atrial fibrillation: executive summary: a report of the American College of Cardiology/American Heart Association Task Force on practice guidelines and the Heart Rhythm Society. Circulation.

[46] Schnabel RB, Yin X, Gona P (2015). 50 year trends in atrial fibrillation prevalence, incidence, risk factors, and mortality in the Framingham Heart Study: a cohort study. Lancet.

[47] Huxley RR, Filion KB, Konety S, Alonso A (2011). Meta-analysis of cohort and case-control studies of type 2 diabetes mellitus and risk of atrial fibrillation. Am J Cardiol.

[48] Larsson SC, Drca N, Wolk A (2014). Alcohol consumption and risk of atrial fibrillation: a prospective study and dose-response meta-analysis. J Am Coll Cardiol.

[49] Bébarová M, Horáková Z, Kula R (2017). Addictive drugs, arrhythmias, and cardiac inward rectifiers. Europace.

[50] Sabzwari SR, Garg L, Lakkireddy D, Day J (2018). Ten lifestyle modification approaches to treat atrial fibrillation. Cureus.

[51] Carter S, Hartman Y, Holder S, Thijssen DH, Hopkins ND (2017). Sedentary behavior and cardiovascular disease risk: mediating mechanisms. Exerc Sport Sci Rev.

[52] Oldgren J, Healey JS, Ezekowitz M (2014). Variations in cause and management of atrial fibrillation in a prospective registry of 15,400 emergency department patients in 46 countries. The RE-LY Atrial Fibrillation Registry. Circulation.

[53] Yuyun MF, Bonny A, Ng GA (2020). A systematic review of the spectrum of cardiac arrhythmias in sub-Saharan Africa. Glob Heart.

[54] Lip GY, Golding DJ, Nazir M, Beevers DG, Child DL, Fletcher RI (1997). A survey of atrial fibrillation in general practice: the West Birmingham Atrial Fibrillation Project. Br J Gen Pract.

[55] Akpa M, Ofori S (2015). Atrial fibrillation: an analysis of etiology and management pattern in a tertiary hospital in Port-Harcourt, southern Nigeria. Res J Health Sci.

[56] Mbakwem AC, Ajuluchukwu JNA, Oke DA (2002). Clinical electrocardiographic and echocardiographic features of atrial fibrillation in Nigerians: an analysis of 39 patients seen at the Lagos University Teaching Hospital. Nigerian Quarterly Journal of Hospital Medicine.

[57] Dokainish H, Teo K, Zhu J (2017). Global mortality variations in patients with heart failure: Results from the International Congestive Heart Failure (INTER-CHF) prospective cohort study. Lancet Glob Health.

[58] Ogah OS, Stewart S, Onwujekwe OE, Falase AO, Adebayo SO, Olunuga T, Sliwa K (2014). Economic burden of heart failure: investigating outpatient and inpatient costs in Abeokuta, Southwest Nigeria. PLoS One.

